# Noncoding RNA Profile in Reovirus Treated *KRAS*-Mutated Colorectal Cancer Patients

**DOI:** 10.3390/diseases11040142

**Published:** 2023-10-16

**Authors:** Rafael Saperstein, Sanjay Goel, Radhashree Maitra

**Affiliations:** 1 Department of Biology, Yeshiva University, 500 W 185th St, New York, NY 10033, USA; rsapers1@mail.yu.edu; 2 Department of Oncology, Rutgers Cancer Institute of New Jersey, New Brunswick, NJ 08901, USA; sanjay.goel@rutgers.edu

**Keywords:** ncRNA, *KRAS*-Mutated Colorectal Cancer, RNA sequencing

## Abstract

Purpose: To investigate the alterations in the expression of noncoding, micro, and small RNA expression during treatment with oncolytic reovirus in *KRAS*-mutated colorectal cancer. Methods: Oncolytic reovirus treatment was administered in phase 1 clinical trial (NCT01274624) for 5 days every 28 days, and blood samples were collected before the administration of the reovirus and 48 h, 8 days, and 15 days after its administration on day 1. Data from the blood samples were sorted using Transcriptome Analysis Software (TAC) 4.0, where a two-tailed *t*-test and a fold change filter were used to ascertain which sample signals had a statistically significant relative fold change of greater than 2 at multiple timepoints before or after oncolytic reovirus administration. Results: The long noncoding RNA’s RP11-332M2.1 (−6.1 x), LINC01506 (−16.18 x), and LINC00534 (−1.94 x) were downregulated at 48 h after reovirus administration [*p* < 0.05]. ncRNA’s EPB41L4A-AS1 (−6.34 x, 48 h; 11.99 x, day 8), JAK2 (2.2 x, 48 h; −2.23 x, day 8), ANXA4 (20.47 x, day 8; −7.54 x, day 15), and PCDH9 (−2.09, day 8; 1.82 x, day 15) were affected by the reovirus treatment and reflected the progress of the treatment [*p* < 0.05]. The small RNA SNORA26 (−1.59 x, day 8) was downregulated 48 h after the reovirus administration [*p* < 0.05]. The microRNA MIR-4461 (6.18 x, day 8; −3.76 x, day 15) was also affected by the reovirus administration [*p* < 0.05]. Conclusion: The administration of oncolytic reovirus to treat *KRAS*-mutated colorectal cancer is reflected in a noncoding RNA profile, and expression levels of the ncRNAs in that profile may thus be able to be used as a potential predictive marker for reovirus-treated colorectal cancer.

## 1. Introduction

As the role of noncoding RNA (ncRNA) has been explored in recent years, the perception of it has changed from being outside the purview of genetic information carriers to being an important part of the RNA transcriptome, which contains microRNA (miRNA) and small nucleolar RNA (sno RNA) and controls different levels of gene expression [1]. ncRNA has been shown to influence the higher organisms’ genomic output and control chromosome architecture, miRNA turnover, and the developmental timing of protein expression, and may also regulate transcription and alternative splicing [2]. ncRNA has also been shown to be a factor in the progression of cancer and tumor proliferation [3].

MicroRNA coding sequences constitute 1–5% of the human genome and regulate at least 30% of protein-coding genes. Over 940 unique miRNA molecules have been studied, and unique functions have been assigned [4]. Different molecules of miRNA have been shown to facilitate or inhibit tumor growth in human cancer. In cancer cells, miRNAs have been found to be heavily dysregulated [5].

Colorectal cancer (CRC) is the fourth most prevalent cancer in the United States and the third most prevalent worldwide [6]. It is the cause of the second-most deaths from cancer both in the United States and worldwide. Current treatments for CRC are chemotherapy, surgery, and radiation therapy. Reovirus treatment, which uses unmodified reovirus to attack the cancer cells, is being considered as an additional treatment, especially for patients with *KRAS*-mutated CRC who are currently considered ineligible to receive epidermal growth factor receptor (EGFR) directed therapy due to resistance and potentially detrimental outcomes [7]. In preclinical models, reovirus was more effective in cells with oncogenic KRAS mutation and has undergone testing in multiple clinical trials, including among patients with CRC that are *KRAS*-mutated [8,9]. It is also known to be useful in treating multiple cancers by decreasing tumor size and promoting tumor necrosis [10]. As a part of a phase 1 clinical trial, we evaluated the efficacy of reovirus treatment in CRC. We have previously documented that reovirus induced autophagy, and there were alterations in noncoding RNA levels [11,12]. Furthermore, we have also shown that reovirus treatment has been shown to induce cellular disintegration at viral factories in the cell, which are empty after the treatment regimen [13]. In this particular study, we focused on the role of noncoding and microRNA by analyzing the alterations in the level of expression in correlation to reovirus treatment.

ncRNA can serve as a biomarker for different types of cancer, influencing and interacting with other ncRNA molecules [14]. The role of ncRNA in regulating both selective and nonselective autophagy has also been explored as more research has been performed on ncRNA in recent years [15].

To further explore the role of ncRNA and miRNA in *KRAS*-mutated CRC, we analyzed 47,841 genes using TAC software to determine statistically significant alteration in gene expression that is associated with apoptosis, cell proliferation, and cancer regulation as a consequence of reovirus treatment. We particularly focused on the ncRNA, miRNA, and other cancer-associated proteins. A total of 17,905 transcripts were identified as noncoding RNAs, including miRNAs and SnoRNAs. Those genes that had a significant *p*-value of 0.05 or below and a fold change of greater than +/−2 were recorded (an increase or upregulation of at least 100% or a decrease or downregulation of at least 50%), resulting in an average of 228 ncRNAs, 57 snoRNAs, and 24 miRNAs meeting that condition. Due to differential expression at each timepoint, there was a slight variation in the number of transcripts that qualified for the cutoff. From those results, the genes that had a fold change of +/−2 and a *p*-value of 0.05 or below at multiple timepoints were tracked across those timepoints, with seven ncRNAs, one snoRNA, and one miRNA being studied.

Three noncoding forms of the transcripts, JAK2, ANXA4, and PCDH9, were marked as noncoding RNAs in our TAC analysis platform. Further analysis revealed that these transcripts were significantly altered in the colorectal cancer microenvironment and, hence, are also included in this study.

Throughout this study, the timepoints representing 8 days and 15 days after reovirus administration are presented as “D8” and “D15”, respectively. 

## 2. Methods and Materials

### 2.1. Patient Selection

Blood samples to source peripheral blood mononuclear cells were harvested from 8 patients. Five patients received reovirus as part of the phase 1 clinical trial (NCT01274624). Three patients were not enrolled in the reovirus-based trial but received equivalent background chemotherapy (i.e., FOLFIRI and bevacizumab). All the patients had *KRAS*-mutated metastatic CRC. 

### 2.2. Ethical Considerations

This study was approved by the ethics committee of Albert Einstein College of Medicine and was performed in compliance with institutional and federal guidelines for clinical research. It was conducted in accordance with the Declaration of Helsinki. 

### 2.3. Institutional Review Board/Ethics Committee Approval and Informed Consent

Based on the Albert Einstein College of Medicine IRB committee-approved consent form (IRB# 07-10-376), all patient blood samples were drawn with informed consent. Following approval by the institutional review board’s ethics committee, 8 patients with KRAS-mutated metastatic colorectal had blood samples drawn. Five of those patients had received reovirus (pelareorep) treatment along with second-line chemotherapy as part of phase 1 clinical trial NCT01274624, and three other patients received equivalent chemotherapy but were not a part of the trial and did not have reovirus (pelareorep) administered. All patients had blood samples drawn at the start of the trial and then at 48 h, 8 days, and 15 days after the start of the trial, with peripheral blood mononuclear cells isolated for transcriptome analysis. Patient samples were deidentified before further processing. 

### 2.4. Sample Harvesting and Processing

Blood samples were harvested from 8 eligible patients into CPT tubes (BD Biosciences, Franklin Lakes, NJ, USA) for the isolation of peripheral blood mononuclear cells. Samples were drawn at various timepoints in relation to the day 1 dose of reovirus—before reovirus treatment and then at 48 h, 8 days, and 15 days after treatment. 

### 2.5. Data Sharing

The authors have shared individual deidentified participant data in the public domain to be available indefinitely. These raw data include peripheral mononuclear cell samples at four timepoints (pre-treatment, 48 h, day 8, and day 15) for each individual involved in the clinical trial. These data can be accessed via the NCBI GEO Accession Viewer (Series GSE173636) using the following URL: https://www.ncbi.nlm.nih.gov/geo/query/acc.cgi?acc=GSE173636 (accessed on 19 August 2023).

### 2.6. Reovirus Administration

The reovirus was supplied by Oncolytics Biotech, Inc. as a translucent-to-clear, colorless-to-light-blue liquid in vials containing 7.2 × 10^10^ tissue culture infective dose (TCID_50_) per mL of reovirus in a phosphate-buffered solution and stored at minus 70 °C. Every 28 days, reovirus was administered as a 60-minute infusion for 5 consecutive days at a tissue culture infective dose (TCID_50_) of 3 × 10^10^/day. 

### 2.7. Transcriptome Analysis

The Transcriptome Analysis was conducted with Transcriptome Analysis (TAC) Software, which uses the genome-guided method to conduct RNA-Seq. TAC 4.0 uses Expression Console^TM^ software (EC) to create probe-level summarization files with cell intensity files (*. CEL), allowing for the initial data quality examination of Affymetrix expression arrays. The total RNA was isolated from the patients’ peripheral mononuclear cell samples and reverse-transcribed using a reverse transcription priming method from an engineered set of primers that exclude sequences that match ribosomal RNA (rRNA). The non-ribosomal RNA-specific primers primed both poly(A) and non-poly(A) mRNA and converted it into double-stranded cDNA, with the first and second-strand enzyme from Kit. The templates were used for in vitro transcription reaction at 37 °C for 16 h to yield cRNA. The ss-cDNA generated from cRNA was chemically fragmented and labeled with biotin and made into a hybridization cocktail using a hybridization kit (Catalog Number 900454) according to the Affymetrix GeneChip protocol and then hybridized to Clariom D gene Chip human probe array. After the array image was generated by a high-resolution Gene Array Scanner 3000 7G (Thermo Fisher Scientific, Santa Clara, CA, USA) and the Cq value for each gene expression of a patient at a given timepoint was recorded, individual sample signals for each patient at each timepoint were extracted from the TAC 4.0 software, organized, and compiled in Microsoft Office Excel. After the results were collected from the patients, the data were uploaded to Transcriptome Analysis Console 4.0.1 (TAC). From those data, ncRNA, small RNA, and miRNA expressions were analyzed at 48 h, 8 days, and 15 days relative to the pre-reovirus administration expression levels in all patients and 8 days relative to 48 h and at 15 days relative to 8 days. 

### 2.8. Statistical Analysis

The patient’s RNA expressions at different timepoints were studied through the use of TAC software. The raw data were fed into the TAC software, which was used to perform a gene expression analysis. Using a two-tailed *t*-test, the data were sorted, leaving only transcripts that were up or downregulated with a significance level of *p* = 0.05 or lower. Following that analysis, the genes were sorted based on fold change, eliminating the genes with a relative fold change of less than 2. The fold change was calculated as the difference between the final value and the initial value divided by the initial value. These genes were then sorted by timepoint using Excel (Microsoft Office 2016) for different combinations of before reovirus administration 48 h after reovirus administration, 8 days after reovirus administration, and 15 days after reovirus administration, and those which had consistent statistically significant fold changes above 2 at different timepoints were studied. 

## 3. Results

**Figure 1:**
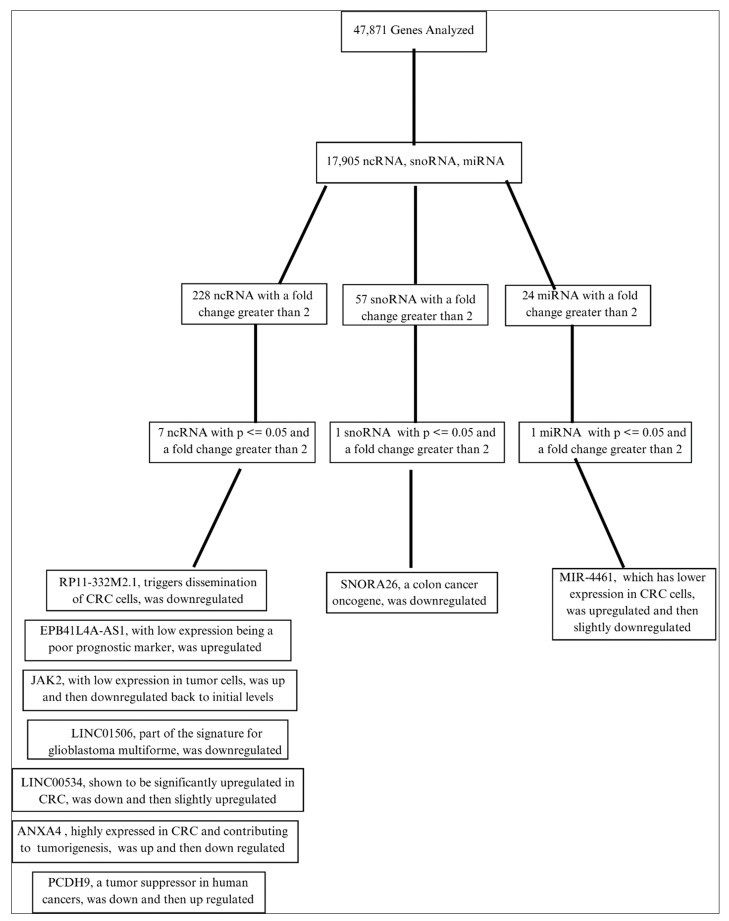
Illustration of our systematic analysis of the functional changes in the ncRNA profile after reovirus administration.

The analysis showed that there were statistically significant changes in the ncRNA profile of *KRAS*-mutated CRC as a result of reovirus treatment, with seven ncRNAs, one miRNA, and one snoRNA having a fold change difference of 2 or greater due to the reovirus treatment (Figure 1). Those changes show support for the efficacy of reovirus administration as a treatment for *KRAS*-mutated CRC and are listed in detail below. Changes without a fold change difference of 2 or greater or without consistent change at different timepoints are listed in Supplementary Table S1.

### 3.1. Downregulation in the Expression of Long Noncoding RNAs after Reovirus Administration

**Figure 2:**
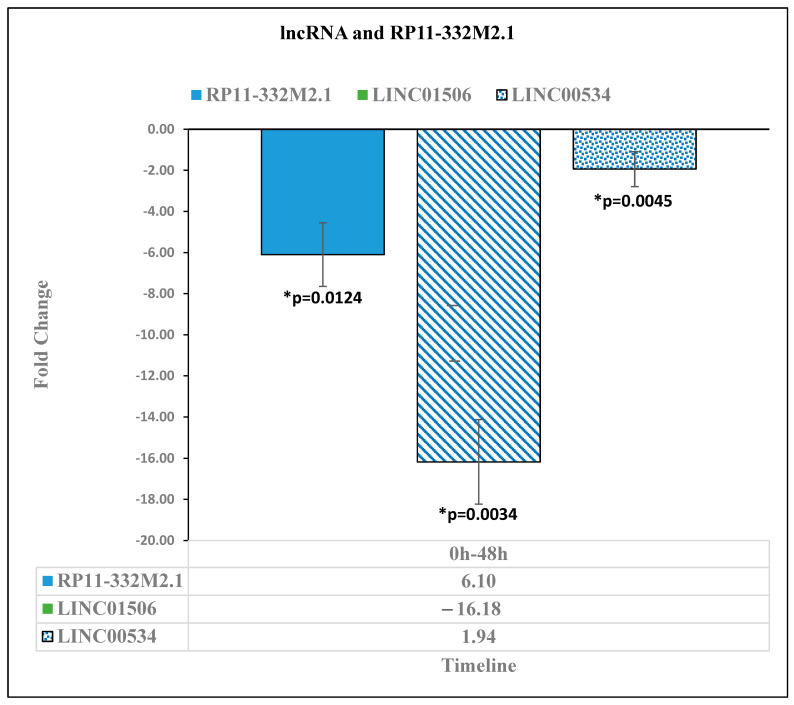
Long noncoding RNA expression level changes between pre-reovirus administration (“0 h”) and 48 h after reovirus administration (“48 h”). An asterisk (*) in the table denotes a significant (*p* ≤ 0.05) fold change.

The lncRNA RP11 has been shown to trigger the dissemination of colorectal cancer cells [16]. In our study, the lncRNA RP11-332M2.1 had a statistically significant (*p* < 0.05) downregulation of 6.1-fold [*p* = 0.0124] at 48 h (Figure 2). As the lncRNA PR11 has been known to have increased expression with increased proliferation of colorectal cancer, the lack of significant fold change in RP11 after 8 days of reovirus administration suggests that RP11-332M2.1 was prevented from further upregulation by the reovirus treatment. Similarly, the lncRNA LINC01506 has been identified as part of a lncRNA signature for glioblastoma multiforme [17]. In our study, LINC01506 was determined to have a statistically significant downregulation of −16.18 [*p* = 0.0034] at 48 h relative to pre-reovirus administration (0 h). Another lncRNA, LINC00534, has been identified as a potential biomarker for colorectal cancer and was significantly upregulated in patients with colorectal cancer [18]. In our study, LINC00534 had a statistically significant downregulation of −1.94 [*p* = 0.0045] at 48 h relative to pre-reovirus treatment (0 h). These downregulations suggest that the reovirus treatment was working to lower the expression of lncRNAs, which are highly expressed in human cancers (Figure 2). 

The lncRNA RP11-332M2.1 expression was downregulated by a −6.1-fold change [*p* = 0.0124] at 48 h after reovirus administration, relative to before reovirus administration. The lncRNA LINC01506 expression was downregulated by −16.18-fold change [*p* = 0.0034] 48 h after reovirus administration relative to its expression level before reovirus administration. The expression of lncRNA LINC00534 was downregulated by a fold change of −1.94 [*p* = 0.0045] at 48 h after reovirus administration, relative to its expression level before reovirus administration. 

### 3.2. Upregulation in the Expression of RNA EPB41L4A-AS1 on Day 8

**Figure 3:**
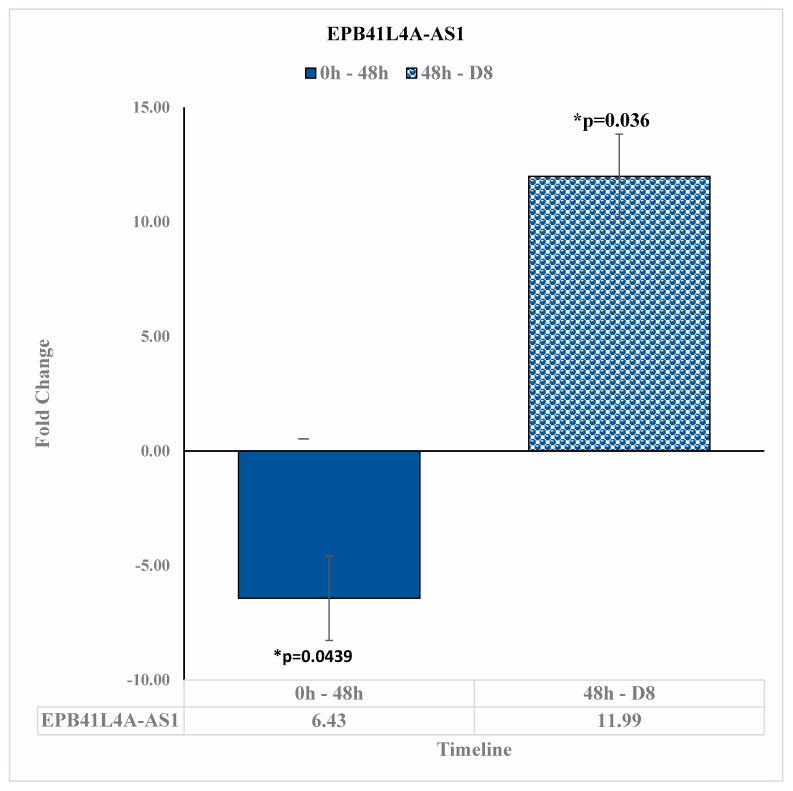
EPB41L4A-AS1 expression changes at pre-reovirus administration (“0 h”), 48 h (“48 h”), and D8. EPB41L4A-AS1 expression is downregulated by −6.34-fold change [*p* = 0.036] at 48 h after reovirus administration, relative to before reovirus administration. Then, EPB41L4A-AS1 expression was upregulated by 11.99-fold change [*p* = 0.0439] 8 days after reovirus administration, relative to the expression levels 48 h after reovirus administration. An asterisk (*) in the table denotes a significant (*p* ≤ 0.05) fold change.

The lncRNA EPB41L4A-AS1 is a repressor of the Warburg Effect [19], and its low expression is related to poor prognosis in human cancers. In our study, EPB41L4A-AS1 was shown to have a statistically significant downregulation of −6.34-fold change [*p* = 0.036] at 48 h relative to before reovirus treatment. At D8, however, EPB41L4A-AS1 was upregulated by the 11.99-fold change [*p* = 0.0439] relative to 48 h (Figure 3). It was not shown to have a significant fold change at D15 relative to D8. This upregulation of EPB41L4A-AS1 suggests that the reovirus treatment took effect at around 8 days after its administration and was associated with greater regulation of glycolysis and glutaminolysis, which are considered a hallmark of cancer metabolism and play a critical role in tumor proliferation [20]. 

### 3.3. Downregulation of Small Noncoding RNA SNORA26 after Reovirus Administration

**Figure 4:**
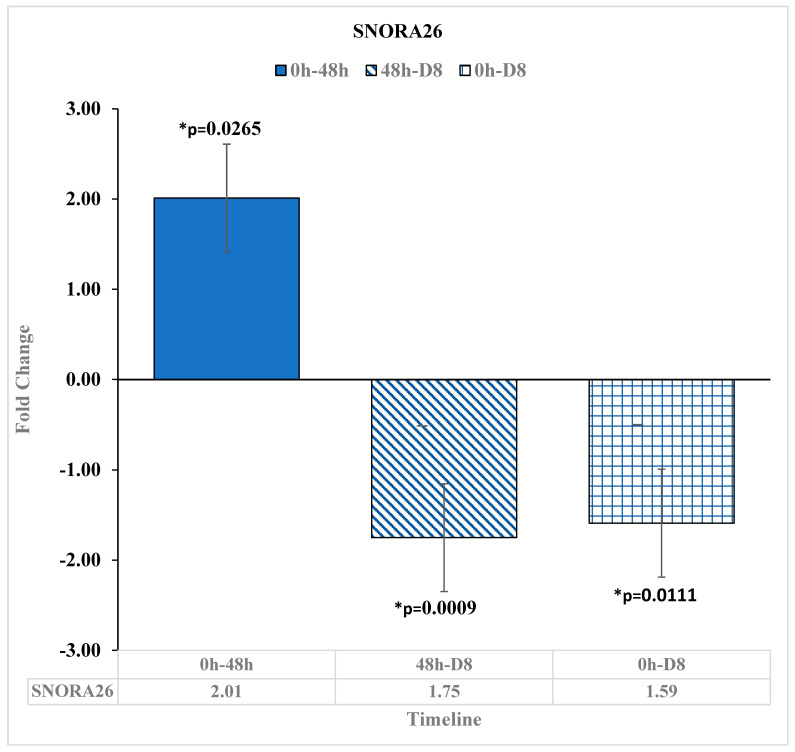
SNORA26 expression changes at pre-reovirus administration (“0 h”), 48 h (“48 h”), and D8. SNORA26 expression was upregulated at 48 h after reovirus administration relative to before reovirus administration, with a fold change of 2.01 [*p* = 0.0265]. Then, SNORA26 expression was downregulated at 8 days after reovirus administration, relative to its expression value at 48 h after reovirus administration, with a fold change of −1.75 [*p* = 0.0009]. Finally, it was downregulated at 8 days after reovirus administration relative to its expression levels before reovirus administration by a fold change of −1.59 [*p* = 0.0111]. An asterisk (*) in the table denotes a significant (*p* ≤ 0.05) fold change.

SNORA26 is a small noncoding RNA that has been shown to act as an oncogene in colon cancers as well as other digestive cancers [21]. In our study, SNORA26 was downregulated from before the reovirus was administered to D8 by −1.59-fold change [*p* = 0.0111] (Figure 4). 

### 3.4. Micro RNA MIR-4461 Shows an Upward Trend in Expression after Reovirus Treatment

**Figure 5:**
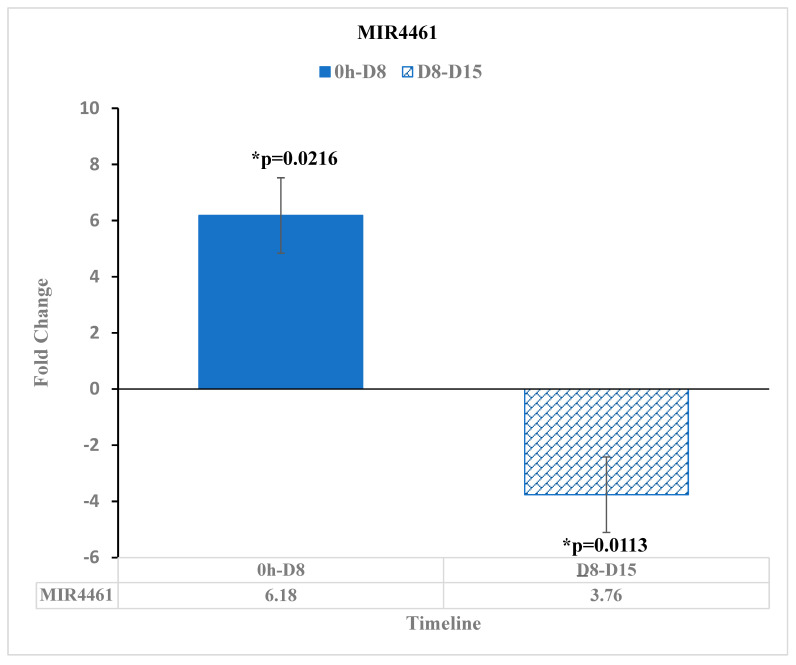
MIR4461 expression changes before reovirus administration (“0 h”), D8, and D15. MIR4461 expression was upregulated by a 6.18-fold change [*p* = 0.0216] at 8 days after reovirus administration relative to its value before reovirus administration. Then, MIR4461 expression was downregulated by a fold change of −3.76 [*p* = 0.0113] at 15 days after reovirus administration, relative to its expression 8 days after reovirus administration. An asterisk (*) in the table denotes a significant (*p* ≤ 0.05) fold change.

MIR-4461 has been observed to have a lower expression in colorectal cancer cells than in normal tissue cells [22]. In our study, MIR-4461 was upregulated by the 6.18-fold change [*p* = 0.0216] at 8 days after reovirus administration relative to before reovirus administration. After this point, MIR-4461 was partially downregulated by a −3.76-fold change [*p* = 0.0113] at D15 relative to D8. This overall upregulation of MIR-4461 suggests that the reovirus treatment was effective at upregulating the MIR-4461 at D8, but the effects may have dropped off as time went on, though not to the level that would normally be observed in colorectal cancer cells (Figure 5).

### 3.5. Expression of JAK2 Is Upregulated Immediately after Reovirus Administration

**Figure 6:**
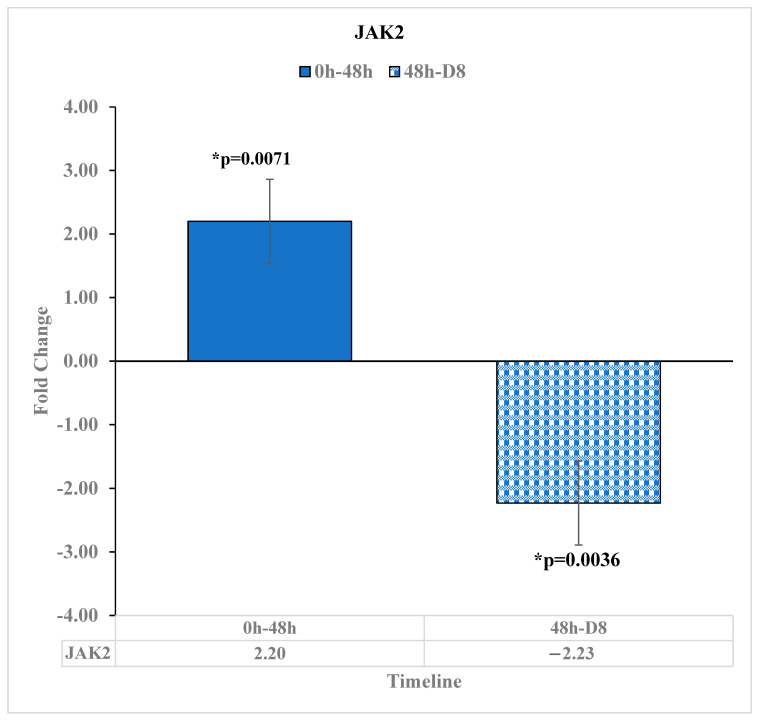
JAK2 Expression changes at pre-reovirus administration (“0 h”), 48 h (“48 h”), and D8. Here, the expression of JAK2 was upregulated by 2.2-fold change [*p* = 0.0071] 48 h after reovirus administration, relative to JAK2 expression before reovirus administration. Then, JAK2 expression was downregulated by −2.23-fold change [*p* = 0.0036] 8 days after reovirus administration, relative to its expression levels 48 h after reovirus administration. An asterisk (*) in the table denotes a significant (*p* ≤ 0.05) fold change.

Mutations in the gene Janus Kinase 2 (JAK2) are present in many forms of cancer [23]. In addition to this, the expression level of JAK2 is correlated with multiple human cancers, with its downregulation in tumor cells relative to normal tissue cells [24]. In our study, JAK2 was upregulated by a 2.2-fold change [*p* = 0.0071] at 48 h relative to before reovirus administration. Then, 8 days after reovirus administration, JAK2 was downregulated by −2.23 [*p* = 0.0036] relative to 48 h. After this point, there was no statistically significant change in the expression of JAK2 at D15 relative to D8. This suggests that the reovirus treatment was in effect around D8, preventing JAK2 from being downregulated, as was seen in other types of proliferating human cancers [24] (Figure 6). 

### 3.6. ANXA4 Is Downregulated at Day 15 after the Reovirus Administration

**Figure 7:**
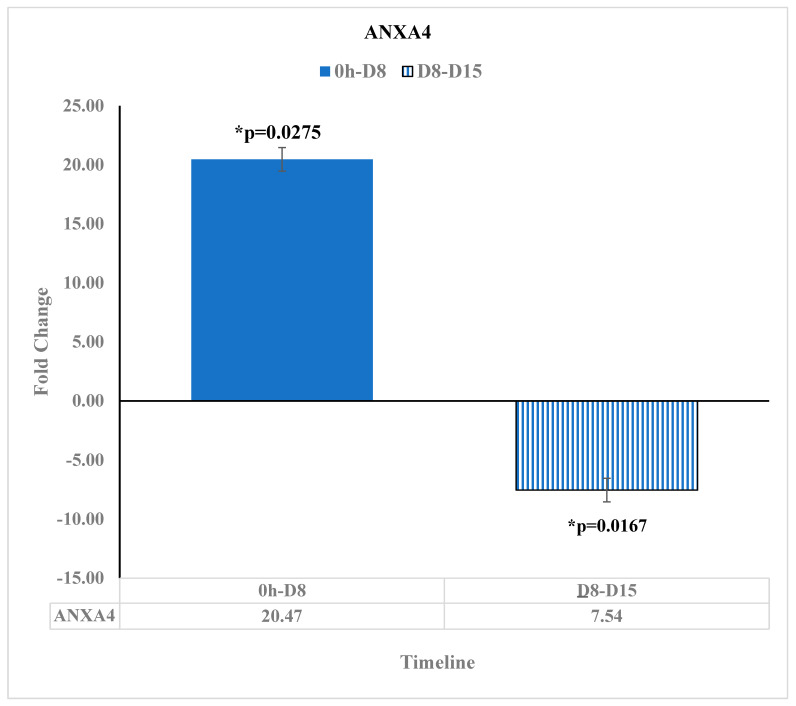
ANXA4 Expression changes at pre-reovirus administration (“0 h”), D8, and D15. ANXA4 expression was upregulated at 8 days after reovirus administration relative to before the reovirus administration, having a fold change of 20.47 [*p* = 0.0275]. Then, ANXA4 expression was downregulated 15 days after reovirus administration relative to its expression 8 days after reovirus administration, having a fold change of −7.54 [*p* = 0.0167]. An asterisk (*) in the table denotes a significant (*p* ≤ 0.05) fold change.

ANXA4, a member of the annexin family, is upregulated in colorectal cancer in addition to enhancing tumor proliferation in human cancers, specifically promoting colorectal cancer tumorigenesis [25]. In our study, ANXA4 was found to be upregulated at D8 relative to before reovirus treatment administration by a fold change of 20.47 [*p* = 0.0275]. However, ANXA4 was downregulated by a fold change of −7.54 [*p* = 0.0167] at D15 relative to D8. This downregulation of ANXA4 after D8 suggests that the reovirus treatment came into effect in downregulating colorectal cancer tumorigenesis at 8 days after administration (Figure 7). 

### 3.7. Tumor Suppressor Protocadherin 9 (PCDH9) Transcript Was Upregulated at Day 15 after Reovirus Treatment

**Figure 8:**
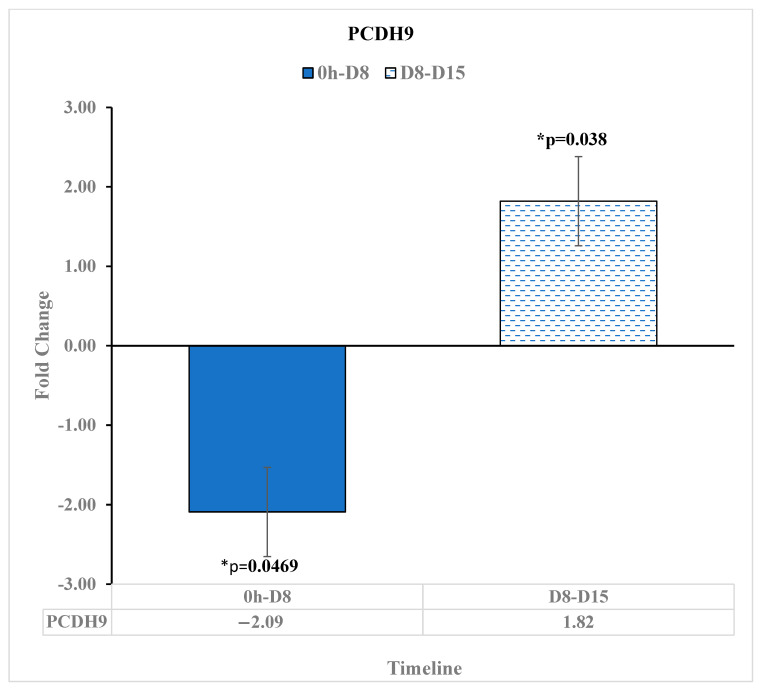
PCDH9 expression changes between pre-reovirus administration (“0 h”), D8, and D15. PCDH9 expression was downregulated 8 days after reovirus administration relative to before reovirus administration, having a fold change of −2.09 [*p* = 0.0469]. Then, PCDH9 expression was upregulated by a 1.82-fold change [*p* = 0.038] at 15 days after reovirus administration relative to 8 days after reovirus administration. An asterisk (*) in the table denotes a significant (*p* ≤ 0.05) fold change.

PCDH9 has been shown to act as a tumor suppressor in human hepatocellular carcinoma [26]. In our study, PCDH9 was downregulated at −2.09-fold change [*p* = 0.0469] at D8 relative to before reovirus administration. Then, PCDH9 was upregulated by a 1.82-fold change [*p* = 0.038] at D15 relative to D8. This suggests that the reovirus treatment came into effect at around 8 days after administration with respect to upregulating tumor suppressors in human cancers (Figure 8).

## 4. Discussion

This study focused on an analysis of the ncRNA, sRNA, and miRNA that were consistently up or downregulated after administration of the reovirus treatment for *KRAS*-mutated colorectal cancer. In order for a transcript to be identified as having been significantly influenced by the reovirus treatment administration over time, it had to meet multiple conditions. First, the transcript had to undergo a fold change of greater than 2 so that smaller and possibly inconsequential changes would not distort our analysis. Then, the changes had to be statistically significant at a level of *p* ≤ 0.05 to ensure statistical significance. Finally, these two conditions had to be met multiple times over different timepoints after the administration of the reovirus treatment in order to identify consistent changes in gene expression. Those timepoints were registered before reovirus administration, 48 h after reovirus administration, and 8 and 15 days after reovirus administration in this study. Then, the transcripts were analyzed so that their function with respect to *KRAS*-mutated colorectal cancer could be identified. Patients who received background chemotherapy did not have statistically significant fold changes in these transcripts, and it was determined that the reovirus treatment influenced the ncRNAs involved in colorectal cancer progression and that those ncRNAs were an important prognostic marker to predict the progression of colorectal cancer within the experimental group of patients. Thus, if the reovirus treatment influenced those ncRNAs that have been shown to aid in combating colorectal cancer with a positive fold change while downregulating ncRNAs that have been shown to promote tumor growth and metastasis, these results can be seen as support for possible efficacy of reovirus treatment of *KRAS*-mutated colorectal cancer. Because the field of ncRNA analysis is a small but growing one, with fewer available references and databases than more established fields, these results can also be used to further their study and provide insight for future studies into the role of ncRNAs. 

The ncRNA analyzed in this study was influenced at around D8 by the reovirus. RP11-332M2.1, a lncRNA that has been observed to trigger dissemination of colorectal cancer cells [16], was initially downregulated by the reovirus before an upregulation of similar magnitude, before not having any significant fold change at the final timepoint. In this way, the reovirus prevented the RP11-332M2.1 from becoming more upregulated and had initially downregulated it by D8.

EPB41L4A-AS1 is a lncRNA that was shown to be a repressor of the Warburg Effect, and its low expression is a poor prognostic marker in human cancers [19]. Initially, EPB41L4A-AS1 was downregulated, but at D8, there was an increase in gene expression. This increase suggests that the reovirus treatment was able to contribute to the regulation of the high rates of glycolysis and glutaminolysis otherwise present in the cancer cells.

JAK2, in addition to having a mutation that is present in many cancers, [23] is downregulated in tumor cells [24]; JAK2 was up and then downregulated by similar magnitudes before not being observed to have statistically significant fold changes. Thus, the reovirus contributed to JAK2 expression levels staying constant to a statistically significant level after D8, in contrast to its demonstrated downregulation in tumor cells.

LINC01506 is a lncRNA and a part of the lncRNA signature for glioblastoma multiforme [17]. Our data show a lesser overall expression of LINC01506 due to the reovirus treatment.

LINC00534 was shown to be significantly upregulated in patients with colorectal cancer and has been identified as a potential biomarker for the disease [18]. Our data showed a decrease and then an increase in gene expression, but not to the extent that it would otherwise be expressed in patients with colorectal cancer due to the reovirus treatment being successful in suppressing LINC00543.

ANXA4 has been highly expressed in colorectal cancer and contributes to its tumorigenesis [19]. In our study, ANXA4 began to have a higher gene expression, but by 15 days after administration of the reovirus treatment, it had a statistically significant negative fold change from 8 days after the reovirus treatment administration. Thus, at around the 8-day mark, the reovirus was effective at downregulating ANXA4 and contributing to the suppression of tumorigenesis in colorectal cancer. 

PCDH9, a tumor suppressor in human hepatocellular carcinoma [26], was upregulated at 15 days after reovirus treatment administration, after being less expressed at 8 days after the treatment. This suggests that around 8 days after administration, the reovirus treatment came into effect in terms of upregulating tumor suppressors that have been found in other human cancers.

SNORA26, an sRNA that acts as an oncogene in colon cancers [21], had lesser gene expression in our study at 8 days after administration relative to before reovirus treatment administration.

MIR-4461 is less expressed in colorectal cancer cells than in normal tissue cells [22]. In our data, MIR-4461 had a statistically significant positive fold change at 8 days after reovirus administration, relative to before the administration of the treatment. Though it had lower expression through day 15, the overall increase in gene expression when it had otherwise been observed to have a lower expression in colorectal cancer cells shows the reovirus having an effect at around 8 days after its administration.

## 5. Conclusions

This study examined the effect of reovirus treatment of *KRAS*-mutated colorectal cancer and if it has an effect on the CRC cell ncRNA and microRNA profile. Our data show that the reovirus treatment indeed has an effect on the ncRNA profile of the cancer cells and that it comes into effect around 8 days after reovirus treatment administration (Table 1). We have reported the alterations of the transcripts with statistically significant and consistent fold changes. The reovirus treatment has downregulated noncoding RNA that has previously been shown to have a high expression in colorectal cancer and kept other genes that have been shown to have a high expression in colorectal cancer unchanged. This interaction with the ncRNA portfolio of human colorectal cancer further confirms that reovirus can not only be utilized as an important therapeutic tool in treating colorectal cancer but also that the ncRNAs studied here can be an important prognostic marker to predict the treatment outcome in colorectal cancer. 

## Figures and Tables

**Table 1:** Table for the values of the fold change in ncRNA, snRNA, and miRNA that may impact colorectal cancer progression at pre-reovirus administration, 48 h, D8, and D15. The *p*-values are shown next to the fold change values at that timepoint.

Noncoding RNA, Small RNA, and microRNA That May Impact Colorectal Cancer Progression—*p*-Values Shown									
RNA Type		Fold Change							
ncRNA		0 h/48 h	*p*-Value	0 h/D8	*p*-Value	48 h/D8	*p*-Value	D8/D15	*p*-Value
	RP11-332M2.1	−6.1	* 0.0124	--	--	6.7	* 0.0013	--	--
	EPB41L4A-AS1	−6.43	* 0.036	--	--	11.99	* 0.0439	--	--
	JAK2	2.2	* 0.0071	--	--	−2.23	* 0.0036	--	--
	LINC01506	−16.18	* 0.0034	--	--	7.49	* 0.0254	--	--
	LINC00534	−1.94	* 0.0045	--	--	3.39	* 0.0003	--	--
	ANXA4	--	--	20.47	* 0.0275	--	--	−7.54	* 0.0167
	PCDH9	--	--	−2.09	* 0.0469	--	--	1.82	* 0.038
snRNA									
	SNORA26	2.01	* 0.0265	−1.59	* 0.0111	−1.75	* 0.0009	--	--
miRNA									
	MIR-4461	--	--	6.18	* 0.0216	--	--	−3.76	* 0.0113

## Data Availability

The authors have shared individual deidentified participant data in the public domain to be available indefinitely. These raw data include peripheral mononuclear cell samples at four timepoints (pre-treatment, 48 h, day 8, and day 15) for each individual involved in the clinical trial. These data can be accessed via the NCBI GEO Accession Viewer (Series GSE173636) using the following URL: https://www.ncbi.nlm.nih.gov/geo/query/acc.cgi?acc=GSE173636 (accessed on 19 August 2023).

## Supplementary Materials

The following supporting information can be downloaded at: https://www.mdpi.com/article/10.3390/diseases11040142/s1, Table S1: Changes without a fold change difference of 2 or greater or without consistent change at different timepoints.
